# Surgical treatment versus observation in moderate intermittent exotropia (SOMIX): study protocol for a randomized controlled trial

**DOI:** 10.1186/s13063-023-07189-0

**Published:** 2023-03-01

**Authors:** Tao Shen, Jingchang Chen, Ying Kang, Daming Deng, Xiaoming Lin, Heping Wu, Jinrong Li, Zhonghao Wang, Xuan Qiu, Ling Jin, Jianhua Yan

**Affiliations:** State Key Laboratory of Ophthalmology, Guangdong Provincial Key Laboratory of Ophthalmology and Visual Science, Guangdong Provincial Clinical Research Center for Ocular Diseases, Zhongshan Ophthalmic Center, Sun Yat-Sen University, Guangzhou, 510060 China

**Keywords:** Intermittent exotropia, Surgery, Observation, Randomized controlled trial, Binocular single vision

## Abstract

**Background:**

Intermittent exotropia (IXT) is the most common type of strabismus in China, but the best treatment and optimal timing of intervention for IXT remain controversial, particularly for children with moderate IXT who manifest obvious exodeviation frequently but with only partial impairment of binocular single vision. The lack of randomized controlled trial (RCT) evidence means that the true effectiveness of the surgical treatment in curing moderate IXT is still unknown. The SOMIX (surgical treatment versus observation in moderate intermittent exotropia) study has been designed to determine the long-term effectiveness of surgery for the treatment and the natural history of IXT among patients aged 5 to 18 years old.

**Methods/design:**

A total of 280 patients between 5 and 18 years of age with moderate IXT will be enrolled at Zhongshan Ophthalmic Center, Sun Yat-sen University, Guangzhou, China. After initial clinical assessment, all participants will be randomized to receive surgical treatment or observation, and then be followed up for 5 years. The primary objective is to compare the cure rate of IXT between the surgical treatment and observation group. The secondary objectives are to identify the predictive factors affecting long-term outcomes in each group and to observe the natural course of IXT.

**Discussion:**

The SOMIX trial will provide important guidance regarding the moderate IXT and its managements and modify the treatment strategies of IXT.

**Trial registration:**

ClinicalTrials.gov: NCT 02736526. Registered April 13, 2016

## Introduction

### Background

Strabismus is a common ophthalmic condition in children with prevalence of 1–5% [1–7], and intermittent exotropia (IXT) in which the eyes intermittently drift outward is one of the most common types of strabismus in childhood [8]. IXT left untreated can result in decreased stereopsis and amblyopia [9] and could eventually lead to intelligence and psychosocial problems [10, 11].

In clinical practice, the strabismus surgery can be performed in order to realign the eyes in severe IXT without binocular single vision (BSV). However, in children with mild IXT who have relatively normal BSV and good control of ocular alignment, the clinicians and parents prefer an observation rather than surgical or conservative treatments [12–14]. The best treatment and optimal timing of intervention for IXT remain unclear [15, 16], particularly for children with moderate IXT who manifest obvious exodeviation frequently but with only partial impairment of BSV. Previous retrospective study showed long-term surgical outcomes in small angle IXT did not appear to be more satisfying than observation [12]. The lack of randomized controlled trial (RCT) evidence means that the true effectiveness of the surgical treatment in curing moderate IXT is still unknown, or even when surgery is the preferred option, there is little agreement on the optimal timing for the surgery in this condition [15, 17, 18]. So, here, in the present study, we conducted a randomized trial of children 5 to 18 years of age with moderate IXT to assess the effectiveness of surgical treatment compared with observation for improving the ocular alignment and stereoacuity over long-term period of 5 years.

### Objectives

The primary objective of the SOMIX trial (NCT 02,736,526) is to compare the long-term cure rate of IXT between the surgical treatment and observation group. The secondary objectives are to identify the predictive factors affecting long-term outcomes in each group and to observe the natural course of IXT.

## Methods

### Study design

**Fig. 1:**
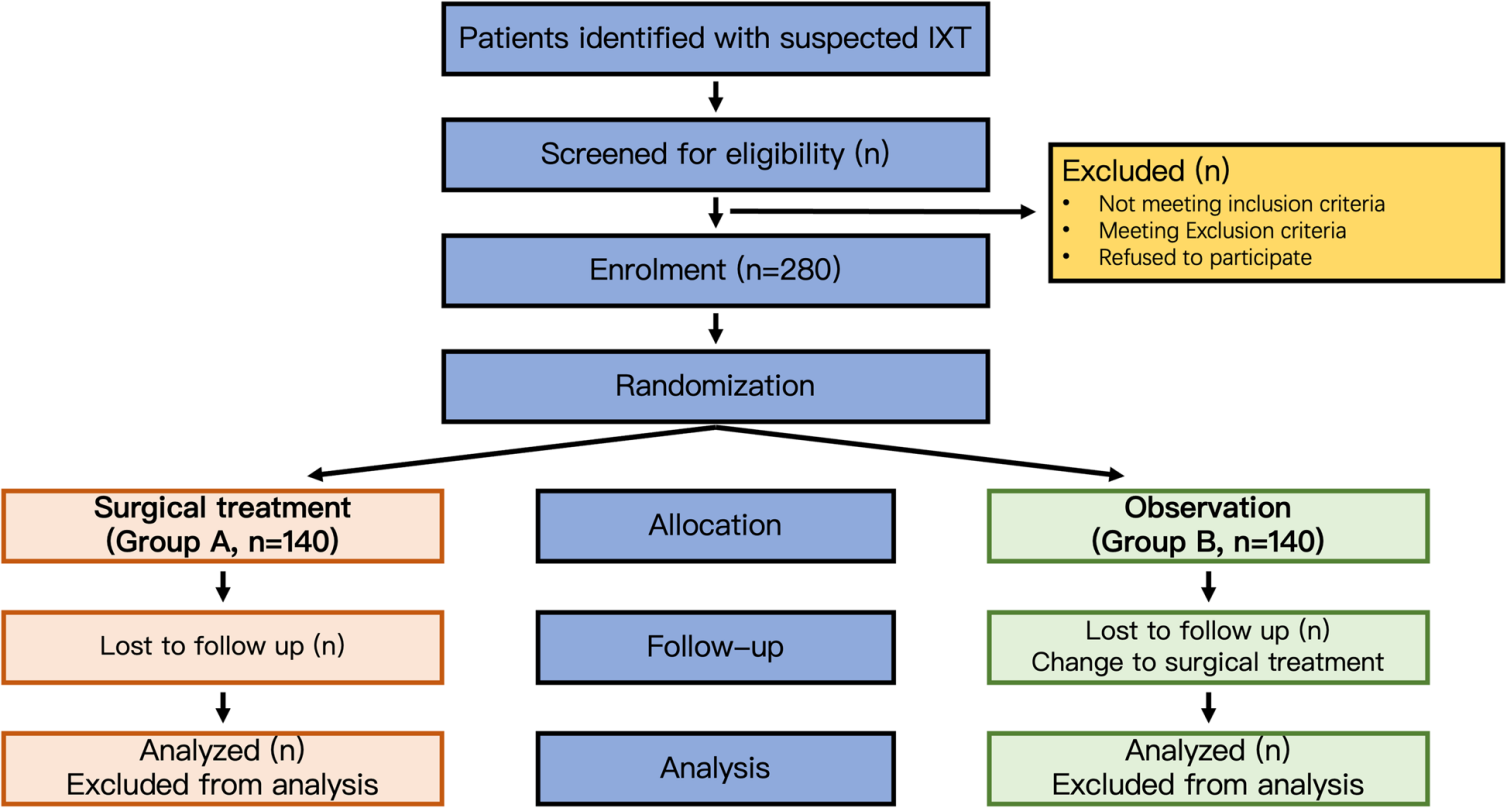
SOMIX trial design

SOMIX is an RCT study to compare the long-term effectiveness of surgical treatment with observation prospectively in patients with moderate IXT (Fig. 1). No patient or public participated in the design of the protocol.

### Study setting

The study setting is the Zhongshan Ophthalmic Center (ZOC), Sun Yat-sen University, Guangzhou, China, which has 11 strabismus specialists and performs about 1000 IXT surgeries per year.

### Participants

The participants are children aged between 5 and 18 years referred to the clinics of ZOC with suspected IXT and subsequently diagnosed as IXT. Prior to any trial-specific procedure, the written informed consent for participation should be obtained from the parents or guardians of the participants.

### Sample size

The sample size for this study was calculated based on a standard two-sided trial with a continuous outcome, and the calculations assume 5% type I error with 80% power. According to an average 30% dropout rate in long-term follow-up period, we anticipate that we will require 280 total participants.

### Clinical tests

The following clinical assessments are undertaken at each visit:

- Binocular single vision (BSV) testing: simultaneous perception, motor fusion, and distance stereopsis using synoptophore; near and distance stereopsis using Titmus test and Randot test
- Best corrected visual acuity (BCVA)
- Measurement of ocular alignment using the prism and alternative cover test (PACT) at near and distance
- Ocular motility examination
- Measurement of control of alignment using revised Newcastle control score (NCS)
- Evaluation of quality of life using Intermittent exotropia questionnaire (IXTQ) [19, 20]
- Cycloplegic refraction
- Routine ocular examination of anterior segment, fundus, and ocular motility

### Eligibility criteria

#### Inclusion criteria

- Age between 5 and 18 years
- Evidence of IXT on the basis of clinical examination: one eye intermittently drifts outward with minimum alternative exodeviation of 15 prism diopters (PD) in the distance; normal ocular motility
- No previous treatment for IXT (including patching, surgery, over-minus lens, vision therapy, and botulinum toxin)
- Presence of simultaneous perception and motor fusion documented using synoptophore but abnormal near and distance stereopsis using Titmus test and Randot test (stereoacuity of > 60 arcsec in the Titmus test or > 63 arcsec in the Randot test is defined as “abnormal stereopsis”)
- BCVA: 0.7 or better at age of 5 to < 6; 0.8 or better at age ≥ 6
- No ongoing or planned amblyopia treatment

#### Exclusion criteria

- Moderate to severe refractive errors: spherical equivalent refraction (SER) >  + 2.00 diopters (D) or <  − 3.00 D (the preexisting refractive errors should be corrected with spectacles)
- Anisometropia > 1.50 D
- Structural ocular pathology
- Significant systemic disorders (for example, neurodevelopmental delay)
- Unable to visit on a regular follow-up (family planning to move out of area)

#### Dropout criteria

- Patients in the intervention group with adverse event or serious adverse event following strabismus surgery
- Patients in the control group with a constant strabismus appears to be developing or whose parents request strabismus surgery

### Recruitment and consent

Children who eligible for the trial are clinically assessed when they attend their initial outpatient visit. Discuss about the management of IXT with the parents or guardians and provide the information about the SOMIX study. Parents or guardians who express an interest and possibility of taking part are then given written information of the SOMIX study. At the recruitment clinic, eligibility is confirmed by checking the results of the initial clinical assessment against the inclusion criteria. The informed consent for participation is obtained from those who decide to participate into the trial.

The following routine clinical assessments are undertaken in order to confirm the eligibility of the participants:

- BSV testing
- BCVA
- Measurement of ocular alignment using PACT at near and distance
- Routine ocular examination of anterior segment, fundus, and ocular motility

### Interventions

- Strabismus surgery (group A)

Surgery is performed by specialized strabismus surgeons (Yan, Deng, Lin, Kang, Chen, Wu, Wang, Qiu, and Shen) according to agreed surgical formulae tailored to the clinical characteristics of the surgeon. Principles involved in the surgical procedure have been agreed as follows:

- General anesthesia
- Bilateral lateral rectus recession to be performed in divergence excessive IXT; unilateral recess/resect surgery to be performed in convergence insufficient IXT; either surgery in basic type of IXT
- Standard sterile preparation of the operative sites
- Conjunctival incisions
- Standard isolation and cleaning of muscle to be operated
- Muscle secured with 6/0 vicryl suture
- Amount of recession and resection assessed on the basis of the maximum distance deviation angle (Table 1), modified according to standard practice of surgeon
- Conjunctival incisions closed with 8/0 vicryl suture
- Antibiotic ointments give at the end of procedure

**Table 1:** Surgical amounts for SOMIX trial

Deviation angle	Unilateral/bilateral recess surgery		Unilateral recess/resect surgery	
**(PD)**	**ULR recession (mm)**	**BLR recession (mm)**	**LR recession (mm)**	**MR resection (mm)**
15	6			
20	8		4	3
25		5.5	5	3.5
30		6	5.5	4
35		6.5	6	4.5
40		7	6.5	5
45		7.5	7	5
50		8	7	6

*PD* Prism diopter, *ULR* Unilateral lateral rectus, *BLR* Bilateral lateral rectus, *LR* Lateral rectus, *MR* Medial rectus

b) Follow-up visit    

**Fig. 2:**
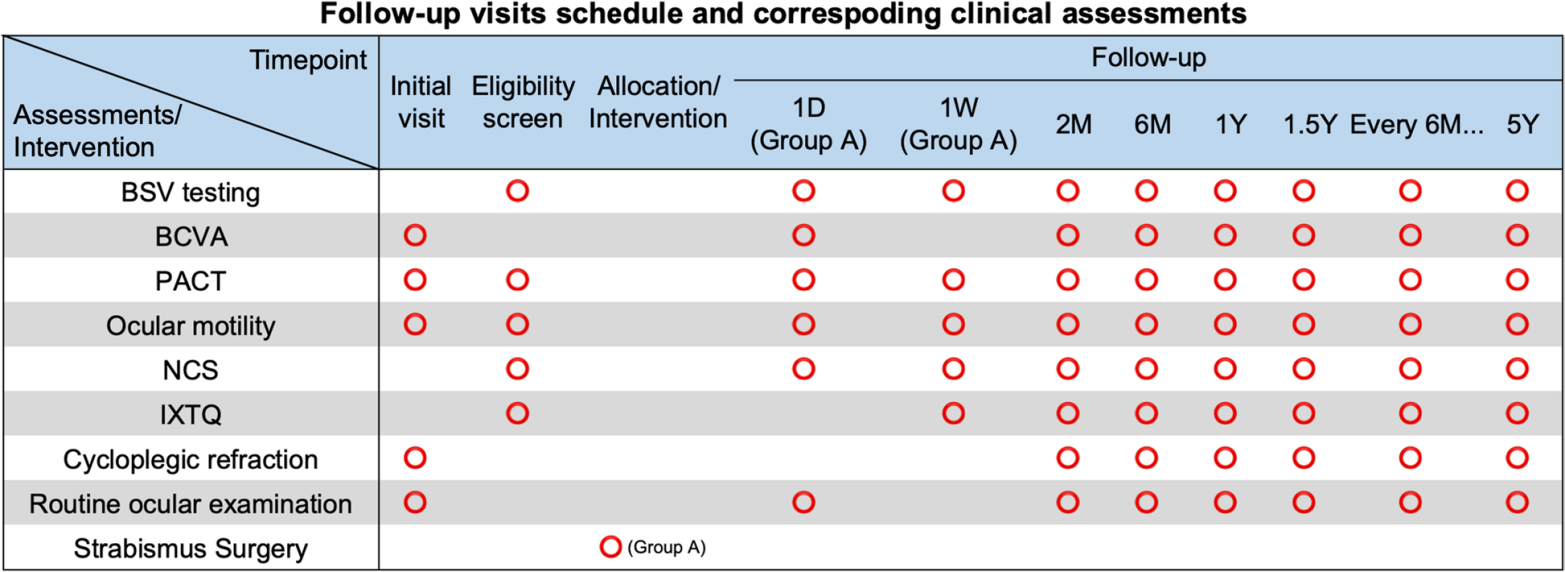
Standard protocol item. The follow-up visits schedule and corresponding clinical assessments. D, day; W, week; M, month; Y, year; BSV, binocular single vision; BCVA, best corrected visual acuity; PACT, prism and alternative cover test; NCS, Newcastle control score; IXTQ, intermittent exotropia questionnaire

The follow-up visits schedule of the study and corresponding clinical assessments for each group are showed in Fig. 2.

Children in the observation group will be offered surgical treatment if a constant strabismus appears to be developing or parents request surgery and the responsible clinical team agrees that this is appropriate.

### Post-trial care

The study will provide post-trial care and insurance for those who suffer adverse events from trial participation.

### Allocation

#### Sequence generation

A blocked allocation (permuted random blocks of variable length) system is being used to randomly allocate patients to the two groups in a 1:1 ratio to intervention (group A) and control (group B) groups.

#### Concealment mechanism

Authorized ophthalmologists followed corresponding treatments for enrolling participants based on grouping information.

#### Implementation

Authorized ophthalmologists will enroll and assign participants. Participants are informed of their group allocation and given the appropriate group allocation information sheet.

### Proposed outcome measures

- Primary outcome

For children with IXT, and their parents, the most relevant outcome from intervention is the restoration of normal ocular alignment, with associated cosmetic and functional benefits. The primary outcome of this RCT study will therefore be the difference in the cure rate of IXT between the surgical treatment and observation group.

Cure of IXT will be defined as:

- Realignment of ocular position at last follow-up, with deviation less than 8 PD both at near and distance
- Improvement of near and distance stereoacuity ≥ 2 octaves (≥ 0.6 log arcsec)

b) Secondary outcome

- Improvement of NCS
- Age-specific evaluation of quality of life (IXTQ)
- Change of refraction

### Data management

#### Data collection

An electronic data capture (EDC) system will be used to collect study data, including baseline characteristics and follow-up clinical evaluations. Participants will be mandatorily followed up regularly in order to access the necessary information.

#### Confidentiality

All data collected in this study will be strictly protected. Participants in the EDC system will be allocated an individual trial identification number to protect confidentiality before, during, and after the trial.

#### Plans to promote participant retention and complete follow-up

Appropriate medical advices and “green channel” of follow-up visit will be provided to participants if they adhere to the protocol and complete follow-up. The trial steering committee will remind the participants the follow-up time in advance through message or phone call.

### Statistical methods

The participant will be included in the analysis, once be randomized, regardless of whether or not the assigned intervention is received, in order to follow the “intention-to-treat” design.

For primary outcome, a chi-squared test will be carried out to evaluate the difference in cure rate at the final assessment between groups. For secondary outcome, a chi-squared test will be used to compare rates of improved NCS outcome between groups; Student’s *t*-tests will be used to assess the validity by matching individual initial and final scores.

Statistical significance was set at *p* < 0.05. Data were analyzed with the SPSS statistical package [SPSS (Statistical Package for the Social Sciences) Inc., Chicago, IL, USA], version 19.

### Potential risks and adverse events

We conduct active monitoring in both groups for potential risks, especially for the observation group for a natural deterioration in their IXT condition. This risk will be managed biannually for the duration of follow-up period, and the treatment will be offered if needed. Adverse event and serious adverse event following strabismus surgery will be recorded and undertaken properly.

### Auditing plan

The ethics committee will meet and communicate important protocol modifications if necessary. Members of the trial steering committee will meet biannually to audit trial conduct.

### Protocol amendments

Any changes to the protocol will be reported to the ethics committee by the trial steering committee. Any modifications from the protocol will be fully documented using a breach report form. The modified protocol will also be synchronized in the clinical trial registry.

### Dissemination policy

The results of the study will be released to the public via publications.

## Discussion

Continued controversy exists regarding IXT and its management, and the natural course of IXT and the factors affecting its control is still unclear. Untreated IXT is traditionally thought to decompensate gradually and that surgical treatment is usually necessary. However, long-term observation indicated that not all surgically untreated IXT deteriorate with passage of time [21–25]. So, there still remains considerable controversy of proper treatment course for IXT, and the indication for and timing of intervention and what therapy is most efficacious remains unclear.

Previous studies have shown that only active observation is a valid course of management for IXT [25, 26]. Surgical treatment is considered for IXT with a progressively increasing deviation angle, increasing deviation frequency, and deterioration of binocular single vision [27, 28]. The younger age at surgery was reported as preoperative factor associated with better surgical outcomes [29, 30]. However, successful motor alignment did not guarantee recovery of BSV [31], and risk of postoperative overcorrection makes the surgical treatment of IXT less favorable [25, 32, 33].

In young patients with moderate IXT who manifest obvious exodeviation frequently but with only partial impairment of BSV, it is quite difficult to make decision for the individual patient whether to choose surgical treatment or not. To the best of our knowledge, no RCT study has been reported to directly compare the surgical treatment versus the natural course of observation in moderate IXT. A pressing need for carefully planned RCT study to provide the evidence for the management of IXT has previously been discussed [34], and a pilot RCT (SamExo) has already been conducted for the feasibility of the full trial [35, 36].

The purpose of SOMIX study is to compare the long-term effectiveness of surgical treatment with observation prospectively in patients with moderate IXT. The main therapeutic goal of intervention is to restore ocular alignment and binocular function, which is also the primary outcome of the present trial. In addition, this study will identify the predictive factors affecting long-term outcomes in each group, which will enhance our understanding of the natural course of moderate IXT, and may modify the treatment strategies of IXT.

## Trial status

Recruitment to the SOMIX trial commences in November 2015 and is ongoing at the time of this manuscript submission. Recruitment of the trial was originally scheduled to end in October 2017, but we have extended this phase up to October 2022 in consideration of low enrollment rate after initial screening. Planned follow-up assessments of both groups are orderly conducted.

## Protocol version

Protocol ID (ZX201304) of December 21, 2021.

## Data Availability

Any data required to support the protocol can be supplied on request.

## Abbreviations

IXT: Intermittent exotropia
RCT: Randomized controlled trial
BSV: Binocular single vision
BCVA: Best corrected visual acuity
PACT: Prism and alternative cover test
NCS: Newcastle control score
IXTQ: Intermittent exotropia questionnaire
PD: Prism diopters
D: Day
W: Week
M: Month
Y: Year
